# Periplasmic binding proteins Bug69 and Bug27 from *Bordetella pertussis* are *in vitro* high‐affinity quinolinate binders with a potential role in NAD biosynthesis

**DOI:** 10.1002/2211-5463.13876

**Published:** 2024-08-08

**Authors:** Leonardo Sorci, Gabriele Minazzato, Adolfo Amici, Francesca Mazzola, Nadia Raffaelli

**Affiliations:** ^1^ Division of Bioinformatics and Biochemistry, Department of Science and Engineering of Matter, Environment and Urban Planning Polytechnic University of Marche Ancona Italy; ^2^ Department of Agricultural, Food and Environmental Sciences Polytechnic University of Marche Ancona Italy; ^3^ Department of Clinical Sciences Polytechnic University of Marche Ancona Italy

**Keywords:** *Bordetella* Uptake Gene, molecular docking, NAD biosynthesis, periplasmic binding protein, quinolinate phosphoribosyltransferase, sequence similarity network

## Abstract

*Bordetella*'s genome contains a large family of periplasmic binding proteins (PBPs) known as Bugs, whose functions are mainly unassigned. Two members, Bug27 and Bug69, have previously been considered potential candidates for the uptake of small pyridine precursors, possibly linked to NAD biosynthesis. Here, we show an *in vitro* affinity of Bug27 and Bug69 for quinolinate in the submicromolar range, with a marked preference over other NAD precursors. A combined sequence similarity network and genome context analysis identifies a cluster of Bug69/27 homologs that are genomically associated with the NAD transcriptional regulator NadQ and the enzyme quinolinate phosphoribosyltransferase (QaPRT, gene *nadC*), suggesting a functional linkage to NAD metabolism. Integrating molecular docking and structure‐based multiple alignments confirms that quinolinate is the preferred ligand for Bug27 and Bug69.

## Graphical abstract

The *Bordetella* genome contains a large family of periplasmic binding proteins (PBPs) called Bugs, whose functions are largely unknown. We show that Bug27 and Bug69 have high affinity for quinolinate, a precursor in NAD biosynthesis. Sequence analysis and molecular docking suggest a link between these proteins and NAD metabolism, highlighting their role in quinolinate uptake.

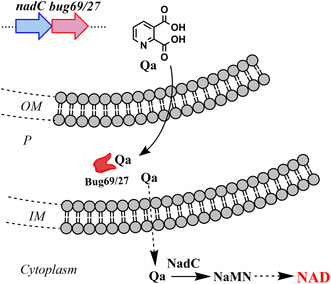

**Abbreviations**
Bug: *Bordetella* Uptake Gene
Ca: citric acid
DHAP: dihydroxyacetone phosphate
Na: nicotinic acid
NAD: nicotinamide adenine dinucleotide
Nam: nicotinamide
NaMN: nicotinic acid mononucleotide
NaMNAT: nicotinic acid mononucleotide adenylyltransferase
NMN: nicotinamide mononucleotide
Pa: picolinic acid
PBP: periplasmic binding protein
PRPP: 5‐phosphoribosyl‐1‐pyrophosphate
Qa: quinolinic acid
QaPRT: quinolinic acid phosphoribosyltransferase
R5P: ribose‐5‐phosphate
TTT: tripartite tricarboxylate transporter

Periplasmic binding proteins (PBPs), also known as substrate binding proteins or extracytoplasmatic solute receptors, bind and deliver specific target molecules, encompassing ions, vitamins, amino acids, carbohydrates, and peptides to a membrane transporter, which, in turn, imports the solute into the cytoplasm. Beyond their canonical role in nutrient uptake, PBPs exhibit versatility as initial receptors, participating in signal transduction and channel gating [1, 2]. Currently, three PBP‐dependent multicomponent transport systems have been documented: (a) the ATP‐binding cassette transporters (ABCs), (b) the tripartite ATP‐independent periplasmic transporters (TRAPs), and (c) the tripartite tricarboxylate transporters (TTTs). ABC systems, categorized as primary transporters, utilize ATP hydrolysis to energize the transport against concentration gradients [3]. In contrast, TRAP and TTT systems facilitate transport by coupling it to an electrochemical gradient, thus functioning as conventional secondary transport systems [4]. An ABC transport system typically comprises a substrate binding protein, a transmembrane (TM) protein, and a nucleotide‐binding protein. Both the membrane and nucleotide‐binding components are usually organized as homo‐ or heterodimers. Such a dimeric structure is crucial for the proper functioning of ABC transporters, enabling coordinated ATP hydrolysis and substrate translocation [5]. Members of the TTT and TRAP families comprise a conserved 12‐TM protein (prototyped by TctA in TTT systems) acting as a symport protein, a 4‐TM protein (TctB) with unknown function, and a PBP protein (TctC) playing the high‐affinity substrate binding role. Recently, the cryo‐EM structure of the sialic acid TRAP transporter SiaQM and the soluble substrate binding “P‐subunit” (SiaP) from *Photobacterium profundum* have been reported, suggesting an ‘elevator‐with‐an‐operator’ transport mechanism where SiaP plays the role of the ‘operator’ of the elevator [6]. TTT transport system is the most recent and least characterized family of transporters using a PBP. Intriguingly, certain bacteria exhibit a redundancy of TctC homologs without apparent membrane counterparts. With 434 *tctT* genes, the genome of α‐proteobacterium *Rhodoplanes* sp. Z2‐YC6860 represents the largest over‐representation of the TTT protein family reported to date, a gene expansion likely spurred by multiple environmental adaptations [7]. In the genome of pathogenic *Bordetella pertussis*, an over‐representation of TctC ‘orphan’ paralogs—referred to as *Bordetella* Uptake Genes (Bug)—has also been observed [8].

Here, we elucidate the role of two orphan TctCs from *Bordetella pertussis*, namely Bug27 and Bug69, as *in vitro* quinolinic acid‐binding proteins. In most bacteria, quinolinate (Qa) is an intermediate of the *de novo* NAD biosynthesis pathway implemented by genes *nadB*, *nadA*, and *nadC* [9, 10]. This pathway leads to the formation of nicotinate mononucleotide (NaMN) intermediate from aspartate and dihydroxyacetone phosphate (DHAP). Once formed, NaMN is converted to NAD by two consecutive reactions [11]. In contrast, most bordetellae lack a fully functional *de novo* NAD biosynthesis pathway, rendering them auxotrophic for the NAD salvage pathway precursors nicotinic acid (Na) and nicotinamide (Nm) (see Ref. [12] and Fig. 1). To meet this nutritional *in vitro* growth requirement, *Bordetella* spp. are conventionally cultured in a standard Stainer Scholte (SS) defined medium supplemented with 30 μm Na (or Nm) [13]. However, Bordetellae, akin to *Streptococcus pyogenes* and other pathogenic bacteria [14, 15], retain a functional terminal component of the *de novo* NAD pathway, the Qa phosphoribosyltransferase (QaPRT) enzyme encoded by gene *nadC*. This enzyme converts quinolinic acid and phosphoribosyl pyrophosphate (PRPP) to NaMN, thus allowing bacterial growth on Qa as the sole NAD precursor [16]. Of significance is the observation of Brickman *et al*. regarding the presence of the gene *bug69* downstream of *nadC* in *Bordetella* species. They hypothesized a direct role of this periplasmic binding protein in the transport of Qa. However, the genetic inactivation of *bug69* in *Bordetella bronchiseptica* did not compromise the bacterium's ability to grow on Qa. This finding led the authors to infer that Bug69 is either not implicated in Qa uptake or that other proteins may be capable of assuming such a function [16]. In *B. pertussis*, a previous study identified Bug27, a paralog of Bug69 sharing 55% sequence identity, as capable of *in vitro* binding to Na and Nam [17].

**Fig. 1:**
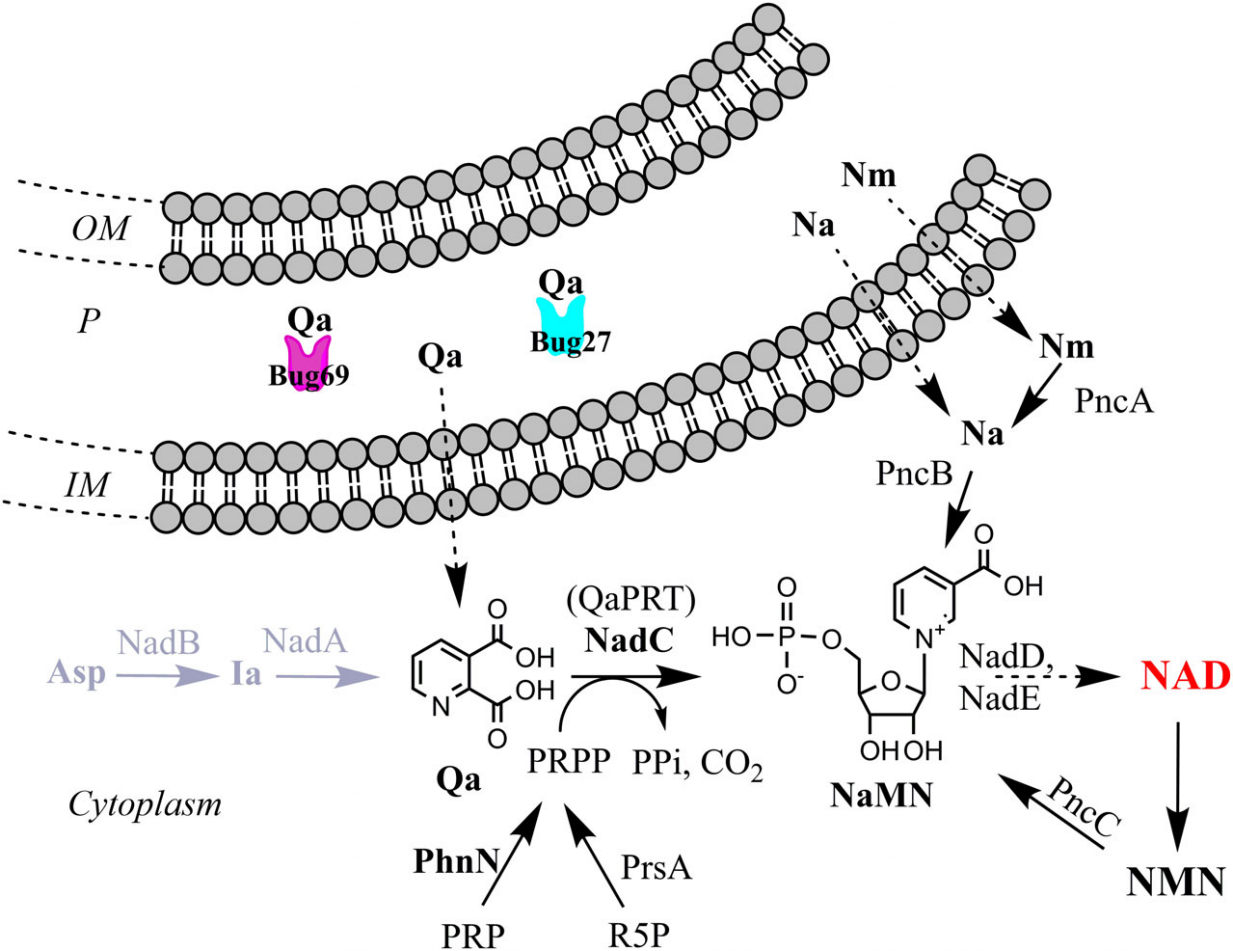
Reconstruction of NAD metabolism in Bordetellae and the proposed role of Bug69 and Bug27 as Qa‐specific solute binding proteins. Pathway reactions are denoted by black arrows. The initial two steps of *de novo* NAD biosynthesis, missing in most Bordetellae, including *Bordetella pertussis*, are depicted in gray. The proposed role of Bug69 and Bug27 as Qa binders is shown. Uptake of pyridines (dotted black arrows) is by unknown mechanisms. Metabolites and enzyme abbreviations (not introduced in the main text). NadD (NaMNAT), nicotinate adenylyltransferase [43]; NadE, NAD synthetase [44]; NMN, nicotinamide mononucleotide; PncC, nicotinamide mononucleotide deamidase [45]; R5P, ribose‐5‐phosphate; PrsA, PRPP synthetase; Other abbreviations: IM, inner membrane; OM, outer membrane; P, periplasm.

The present study demonstrates for the first time that *Bordetella pertussis* Bug69 and Bug27 are *in vitro* high‐affinity Qa binders. This experimental result is supported by a comprehensive evolutionary and *in silico* structural analysis. Finally, comparative genomics revealed a potential functional association of these orphan Bugs with a quinolinate salvage pathway for NAD biosynthesis in Bordetellae and other bacterial species.

## Experimental procedures

### Cloning, expression, and purification of Bug69 and Bug27

Genes were amplified from the genomic DNA of *Bordetella pertussis* Tohama I for cloning into pET28c vector. For *bug27*, the forward and reverse primers were designed as 5′‐TATCCATGGTTGCCACCGGCGACTTCCCG‐3′ and 5′‐GCCAAGCTTGTCCACTTTCAGGCCGATCTGC‐3′ to introduce *Nco*I and *Hind*III restriction sites, respectively. For *bug69*, primers were 5′‐TATCCATGGTTGCGCCGGCCACCTATCCC‐3′ and 5′‐AAACTCGAGGTCGGCGCAGCCCCA‐3′ carrying *Nco*I and *Xho*I sites, respectively. Constructs were used to transform BL21(DE3) competent *Escherichia coli* cells for protein expression. Cells were grown at 37 °C in Luria Bertani medium supplemented with kanamycin (50 μg·mL^−1^). After reaching an OD_600_ of 0.7, the cultures were shifted at room temperature for 20 min, and protein expression was induced with 1 mm isopropyl b‐d‐1‐thiogalactopyranoside. After 15 h of induction, cells were harvested by centrifugation at 5000 ***g*** for 8 min at 4 °C. All the following steps were performed at 4 °C. The pellets were resuspended in one‐twentieth of the original culture volume of lysis buffer (50 mm sodium phosphate, pH 8.0, 0.3 m NaCl, 5 mm imidazole) freshly supplemented with 1 mm phenylmethylsulfonyl fluoride and protease inhibitor cocktail. Cells were disrupted by passage through a French pressure cell (18 000 psi), and crude extracts were clarified by centrifugation at 20 000 ***g*** for 25 min at 4 °C. Both His‐tagged Bug proteins were purified by NiNTA affinity chromatography. The clarified supernatants were loaded onto 1 mL NiNTA columns (Qiagen) equilibrated with the lysis buffer. After washing with 20 mm imidazole in the same buffer, recombinant proteins were eluted with an imidazole concentration of 250 mm. Purification was monitored by SDS/PAGE, according to Laemmli [18]. Protein concentration was determined by Bradford's method [19], and about 9 mg of pure Bug27 and 5 mg of pure Bug69 were obtained starting from 250 mL of cultures.

### Fluorescence‐based thermal shift binding assay

The assay was adapted from a previously published method [20], and analyses were performed after proteins' denaturation and renaturation steps to remove endogenous ligands, as described for Bug27 [17]. Briefly, the assay was carried out in a RotorGene 3000 real‐time PCR in a final assay volume of 25 μL. The assay mixture contained 1 μm of proteins, different concentrations of ligands (dissolved in water), and 2.5× Sypro orange (Invitrogen, Waltham, MA, USA) in 40 mm Tris/HCl, pH 8.0, 250 mm NaCl. The mixtures were pre‐heated at 35 °C for 5 min, then heated to 40 °C for 15 s, and finally heated from 40 to 99 °C with a heating rate of 0.1 °C·s^−1^. The fluorescence intensity was measured with Ex/Em: 470/585 nm. The melting temperature (*T*_m_) was determined by taking the maximum point of the slope‐derivative peak.

### Intrinsic tryptophan fluorescence quenching assay

The dissociation constants (*K*_D_) of Qa for Bug27 and Bug69 were determined using tryptophan fluorescence titration. The fluorescence quenching was carried out in a cuvette with a path length of 1 cm. The Bug proteins were prepared following the same procedure as for the thermal shift assay, with a final concentration of 0.1 μm. The intrinsic fluorescence spectra of Bug27 and Bug69 were recorded by a fluorescence spectrophotometer (Perkin Elmer LS‐45, Waltham, MA, USA) at 20 °C using excitation at 295 nm and emission ranging from 290 to 450 nm. Notably, Qa itself induced negligible changes in fluorescence under the same experimental conditions. Tryptophan fluorescence spectra were collected before and after titration with varying concentrations of Qa. The dissociation constant of Qa to Bug27 and Bug69 was determined by fitting the normalized fluorescence intensity at 328 nm using the following equation [21]:

$$f=\left[\left(\mathrm{Pt}+\mathrm{Lt}+K_{D}\right)-\sqrt{{\left(\mathrm{Pt}+\mathrm{Lt}+K_{D}\right)}^{2}-4\text{PtLt}}\right]/2\mathrm{Pt}$$

where *f* is the fractional change, *K*_D_ is the dissociation constant, and *Pt* and *Lt* are the total concentration of protein and Qa, respectively.

### Statistical analysis

Statistical analyses were performed using prism 7 (GraphPad software, San Diego, CA, USA). The thermal shift binding assays and the tryptophan quenching assays were performed in triplicate. The *K*_D_ values ± Std. Error are determined using nonlinear regression analysis as reported above.

### Bug69‐Bug27 sequence similarity network

We used the “Sequence BLAST or option A” within the EFI‐Enzyme Similarity Tool (available at https://efi.igb.illinois.edu/efi‐est/index.php) to perform all‐by‐all blast analysis of protein homologs of query Bug69 *from Bordetella pertussis* Tohama I (Uniprot ID: Q7W019). Applying the default cutoff *e*‐value of 10^−5^ for Uniprot BLAST retrieval and SSN edge calculation, we limited the retrieval of homologs to a maximum of one thousand (default option). This dataset contained 928 unique sequences. The full network 10^−120^ SSN was generated by applying an *e*‐value cutoff of 10^−120^ to the full network. Ninety‐four paralogs of the TctC family are present in *Bordetella pertussis*. With these stringent search parameters, we collected a set of Bug69 homologs, including Bug27, the closest paralog in *B. pertussis* with a sequence identity of 59%, but excluded all other more distant paralogs. An 85% identity Representative node network was visualized on the Cytoscape platform [22], *i.e*., all connected sequences that share 85% or more identity are grouped into a single meta node.

### *In silico* binding analysis and other bioinformatics tools

Visualization, molecular docking, 2D and 3D ligand‐pocket interaction analysis were performed with tools integrated in Molsoft ICM‐PRO 3.9‐2c [23]. The X‐ray structure of Bug27 (PDB code: 2QPQ) served as the template for docking pyridine derivatives. Prior to docking, the protein was prepared by removing waters, optimizing the hydrogen bonding network, and the orientation of His, Pro, Asn, Gln, and Cys residues. We used the ICM Pocket Finder algorithm [24] to identify the ligand binding pocket of Bug27 (PDB: 2qpq). One of the identified pockets, which partially overlapped with the co‐crystallized citrate ligand, was used as the template for docking simulations. The co‐crystallized citrate ligand was removed before docking. In independent docking tests, confidence in the docking pose was achieved by progressively increasing the simulation length (using the ‘Effort’ parameter, ranging from 1 to 10 in ICM‐PRO) until convergent results were obtained. Structure‐based multiple sequence alignments were generated with PROMALS3D [25] and rendered with Espript 3.0 [26].

## Results

### Sequence similarity networks of *B. pertussis* Bug69 and Bug27

**Fig. 2:**
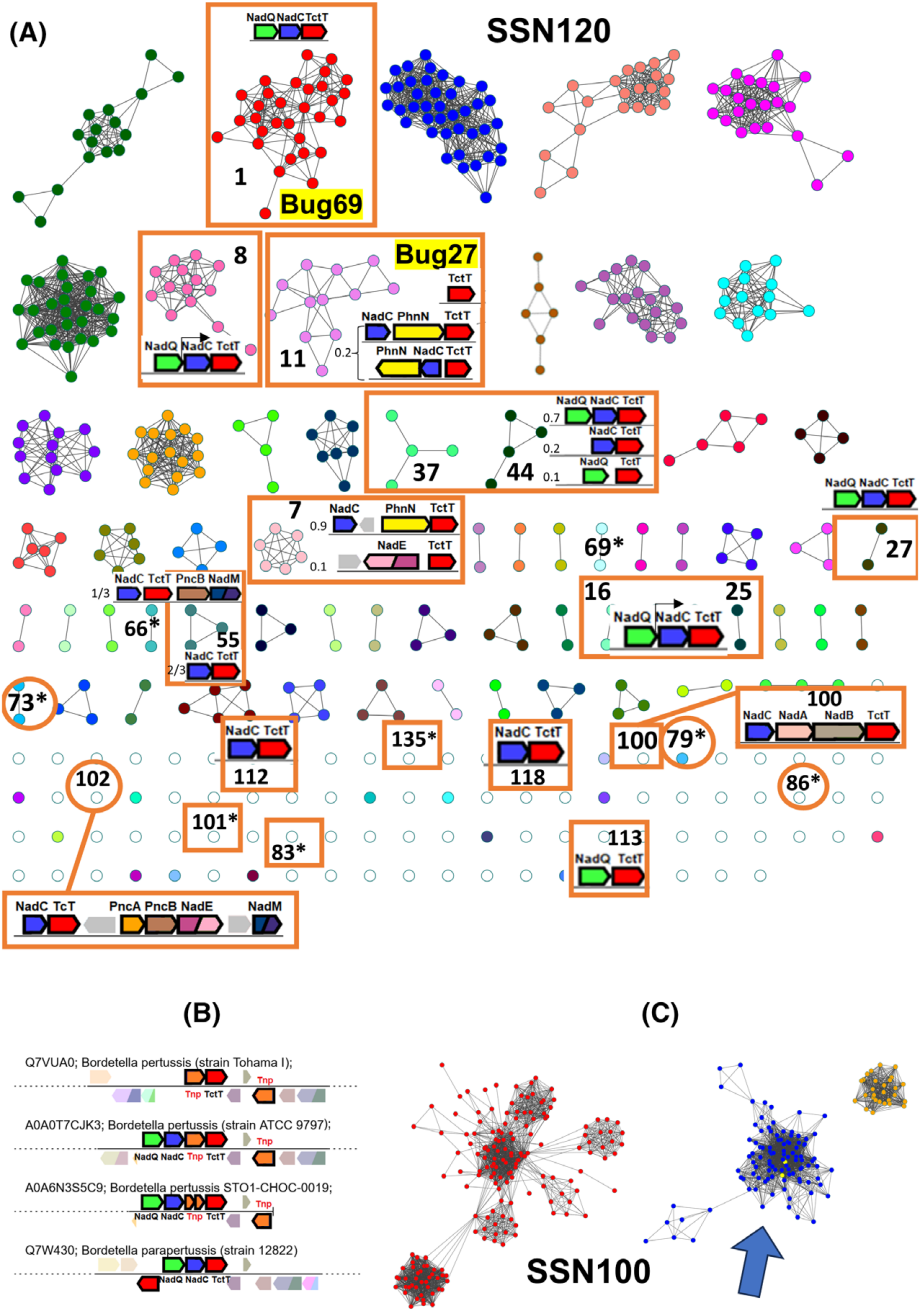
Sequence similarity networks and genome context analysis of *Bordetella pertussis* Bug69 and Bug27 homologs. (A) SSN displayed at an alignment score of 120 (~ 67% sequence identity) generated with *B. pertussis* Bug69 (Uniprot: Q7VUA0) as the input sequence. Clusters with the TctC homolog in a NAD cofactor‐related genomic context are boxed, and the gene cluster is shown inside. The relative frequency is indicated on the left when different gene contexts are present within the same SSN cluster. Clusters with an asterisk possess the same gene context as in 1 (not repeated for clarity). Taxonomy of sequences in NAD‐related clusters: *Bordetella* spp., *Achromobacter* spp., and *Alcaligenes xylosoxydans* (1); *Cupriavidus* spp. (7); *Comamonas* spp. (8); *Bordetella* spp., *Variovorax* spp., *Jezberella montanilacus*, *Alcaligenes faecalis*, *Achromobacter pestifer* (11); *Delftia* spp. (16); *Algalinigenes* spp. (25); *Orrella dioscoreae*, *plant metagenome* (27); *Pollutimonas* spp., *Cupriavidus* sp. *USMAA2‐4*, *Alcaligenaceae bacterium* (37); *Pseudorhodoferax* spp., *Lampropedia cohaerens* (44); *Vandammella animalimorsus*, *Franklinella schreckenbergeri* (55); *Alcaligenaceae bacterium* (66, 69, 73, 79); *Pusilimonas* sp. *ANT_WB101* (79); *Pseudorhodoferax* sp. *Leaf267* (83); *Comamonas kerstersii* (86); *Comamonas* sp. *BIGb0124* (113); *Lampropedia hyalina DSM 16112* (100); *Paenalcaligenes hominis* (101); *Verticiella sediminum* (118); *Pusillimonas* sp. *TS35* (135). (B) Genome neighborhood diagrams of *B. pertussis* strains show transposable elements in the context of TctC. (C) A partial view of the SSN is displayed at an alignment score of 100. The blue cluster contains all NAD‐related nodes (boxed) of SSN120.

To investigate the sequence‐function relationship of the two protein families typified by *B. pertussis* Bug69 and Bug27, we adopted an integrated strategy leveraging sequence similarity networks (SSN) and genome context analysis tools available at https://efi.igb.illinois.edu/ [27, 28, 29]. Sequence similarity networks visually represent pairwise sequence relationships within clusters of homologous proteins [30]. In these networks, each node represents a protein, and edges connect pairs of nodes with pairwise relationships better than a user‐defined threshold. These pairwise relationships correspond to BLAST alignment associated with an *E*‐value. When properly selected cutoff values are applied, proteins with identical functions tend to segregate into distinct clusters. Using Cytoscape, we examined the SSN for 928 top homologs of *B. pertussis* Bug69 (Option A in the EFI‐EST tool) at an *e*‐value of 10^−120^, which corresponds to a median sequence identity of approximately 67%, yielding a total of 156 discrete clusters (Fig. 2A). Bug27 is a closely related paralog of Bug69, with a 59% identity. Hence, this approach also allowed for a comprehensive retrieval of Bug27 homologs. Bug69 and Bug27 were located in separate clusters, while the remaining 92 “Bugs” from *Bordetella pertussis* were excluded from the initial dataset because they were more divergent (refer to the Methods sections for parameters used for generating the initial dataset of sequences).

### Genome context analysis and phylogenetic distribution of Bug69 and Bug27

To aid functional inference, we integrated the SSN with genome neighborhood information using the EFI‐GNT tool (Fig. 2A) and assessed the overall phylogenetic distribution of Bug69 and Bug27 homologs. Our analysis revealed that in all 80 members of the Bug69‐containing cluster, almost equally represented by *Bordetella* and *Achromobacter* spp. of the Alcaligenaceae family, *bug69* is in an operon downstream of the NAD transcriptional regulator *nadQ* [31] and *nadC* (Cluster 1 in Fig. 2A). The only exception is *B. pertussis* Tohama I, where chromosomal rearrangement has translocated the gene elsewhere (Fig. 2B). A close inspection of the gene neighborhood in all sequences showed a prevalent association of TctC with *nadC* and *nadQ* in many other clusters populated by genera of Alcaligenaceae, Comamonadaceae, and Burkholderiaceae (Fig. 2A). Occasionally, *tctC* is adjacent to only *nadC* or *nadQ*, with the third gene located elsewhere. In addition to their physical proximity, genes *nadC* and *tctC* are also predicted to be co‐regulated by NadQ transcriptional regulator as shown for the examples of *Delftia acidovorans* SPH‐1 (Cluster 16 in Fig. 2A) and *Comamonas testosteroni* KF‐1 (Cluster 8) (https://regprecise.lbl.gov/index.jsp). Overall, our comprehensive analysis corroborates and extends the functional association of QaPRT with Bug69, suggesting that Bug69 might have evolved to recruit the QaPRT substrate quinolinic acid.

*Bordetella pertussis* Bug27 is found in cluster 11 of the SSN (Fig. 2A). The structural and functional characterization of *B. pertussis* Bug27 showed its capacity to bind Na and Nam, two precursors of NAD, despite not being in a genome context with NAD metabolic genes [17]. However, an inspection of the gene neighborhood of orthologs from *Variovorax* spp. (Alcaligeneaceae) and the more distant *Jezberella montanilacus* (Comamonadaceae) shows that *bug27* is adjacent to *nadC* and *phnN*. The latter gene encodes the enzyme ribose 1,5‐bisphosphate phosphokinase, which converts ribose 1,5‐biphosphate (PRP), a crucial component in the metabolism of ribonucleotides and phosphonates, into phosphoribosyl pyrophosphate (PRPP) [32, 33]. PRPP, in turn, serves as the phosphoribosyl donor in the QaPRT‐catalyzed reaction (see Fig. 1). This finding suggests a role for Bug27 in NAD metabolism as an alternative binder of quinolinic acid, distinct from Bug69.

Furthermore, our gene neighborhood analysis has revealed, in more distant species, alternative genetic arrangements of TctC homologs with the *de novo nadBAC* genes, salvage *pncAB* genes, and downstream NAD biosynthesis genes *nadE* or *nadM* (see clusters 7100, 102). This provides additional evidence of the involvement of Bug69 and Bug27 in NAD metabolism and allows us to formulate a hypothesis on the evolutionary scenario of NAD‐related Bug proteins. These proteins could have initially emerged as promiscuous binders of NAD precursors as Qa, Na, Nm, and Asp and subsequently specialized into more specific Qa scavengers under selective pressure.

Lastly, applying a more relaxed alignment score threshold of 100, we generated a sequence similarity network in which all TcTs found in NAD metabolic contexts in SSN120 segregate in a single, allegedly isofunctional cluster (Fig. 2C).

### *In vitro* characterization of *B. pertussis* Bug69 and Bug27

**Fig. 3:**
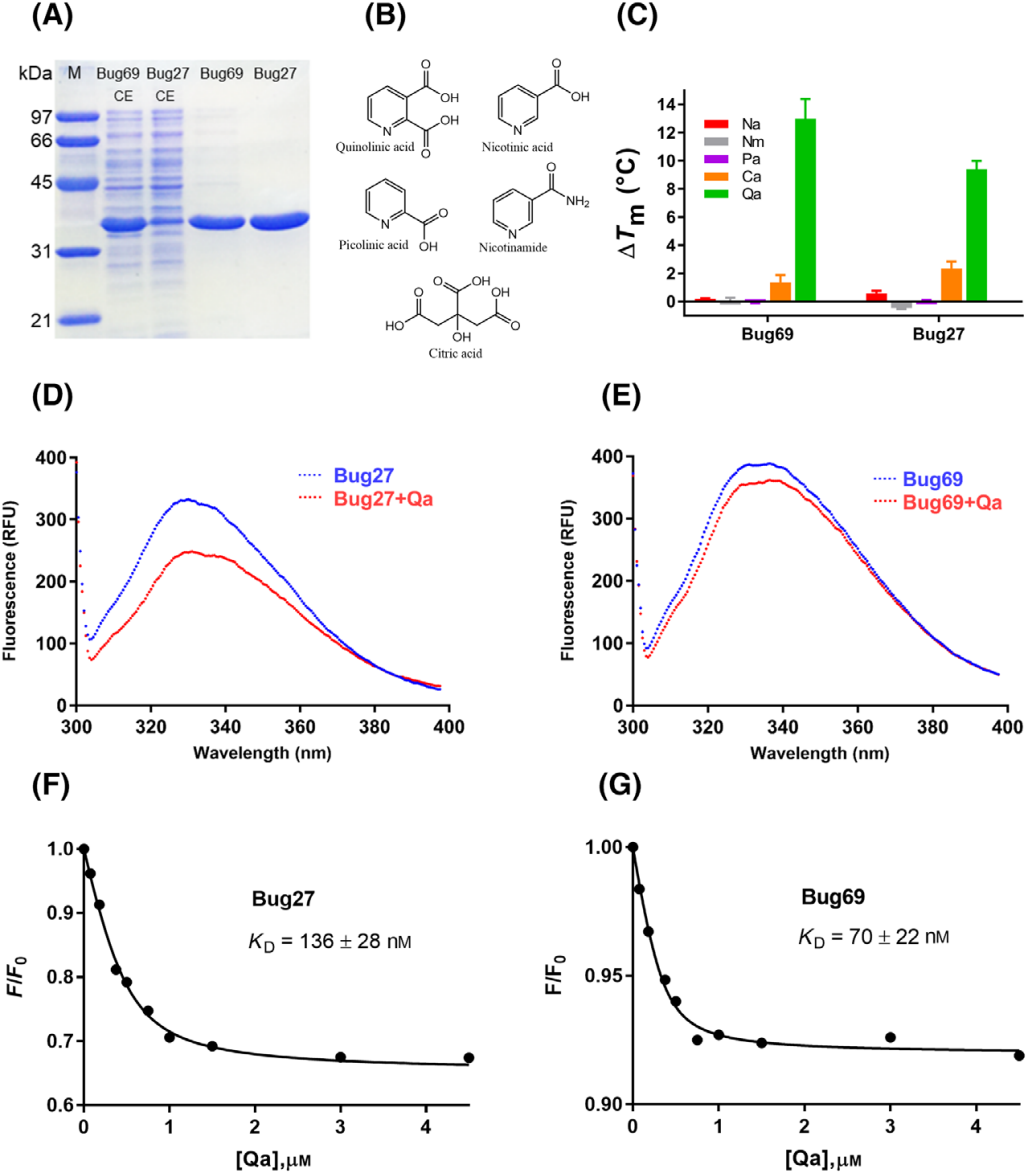
*In vitro* characterization of *Bordetella pertussis* Bug69 and Bug27. (A) SDS/PAGE of purified Bug69 and Bug27. M, protein molecular marker; CE, crude extracts (CE). (B) Chemical structures of pyridine NAD precursors and other carboxylic compounds used for the *in vitro* binding screening. (C) Histograms of the Δ*T*_m_ (°C) of Bug69 and Bug27 in the presence of 1 mm concentration of the tested compound. The data represent the mean ± SD of three independent experiments. (D, E) Fluorescence emission spectra of Bug27 and Bug69 in the presence and absence of saturating quinolinate (4.5 μm). The excitation and emission wavelengths were 295 and 328 nm, respectively. (F, G) The amplitude of normalized fluorescence as a function of quinolinate concentration for Bug27 (left) and Bug69 (right). The solid line represents a fit to the equation provided in the methods section. Data represent the mean of three independent experiments.

As inferred from our bioinformatics analysis, the most plausible hypothesis is that Bug69 and Bug27 may be involved in the transport of Qa. To experimentally validate this conjecture, we first obtained Bug69 and Bug27 in recombinant form and purified them to homogeneity (Fig. 3A). We then employed a fluorescence‐based thermal unfolding assay [20] to test the NAD precursors Qa, Na, and Nm as potential ligands (Fig. 3B). We included the pyridine picolinic acid (Pa) and tricarboxylic citric acid (Ca) as control molecules. Ca was previously co‐crystallized with Bug27 in an open state, thereby being identified as a *non‐bona* fide ligand of Bug27 [17]. An initial screen at a fixed concentration of 1 mm showed that, among the tested compounds, only Qa significantly affected the thermal stability of the proteins (ΔTm), with a > 50‐fold preference over Na in Bug69 and about 20‐fold in Bug27. On the other hand, Ca induced modest shifts, around 10 and 5 times less than Qa, in Bug69 and Bug27, respectively (Fig. 3C). Both proteins feature a conserved Trp alongside varying quantities of tyrosines and phenylalanines, hinting at the potential utility of fluorescence spectroscopy in accurately evaluating quinolinate ligand binding. A comparison of the fluorescence emission spectra of Bug27 and Bug69 in the absence and presence of quinolinate showed a significant fluorescence quenching (Fig. 3D,E). However, this effect differed in magnitude for each protein, indicating slightly distinct changes in the tryptophan environment upon ligand binding. Tryptophan emission peaked at 330 nm (excited at 295 nm) for Bug27 and at 335 nm for Bug69, with the peak emission wavelength remaining unchanged upon quinolinate binding. Based on quinolinate‐induced fluorescence change, the quantitative analysis revealed that the dissociation constants (*K*_D_) for quinolinate binding to Bug27 and Bug69 were 136 ± 28 nm and 70 ± 22 nm, respectively (Fig. 3F,G). These results suggest that Bug69 and Bug27 proteins are high‐affinity quinolinate binders. The affinity of Qa for Bug27 was about three times higher than that of Na, Nm, and Ca, as previously calculated with the same approach [17]. Hence, using two independent biophysical assays, we experimentally supported our bioinformatics prediction that Qa could be the natural ligand of both Bug69 and Bug27.

### *In silico* binding analysis

**Fig. 4:**
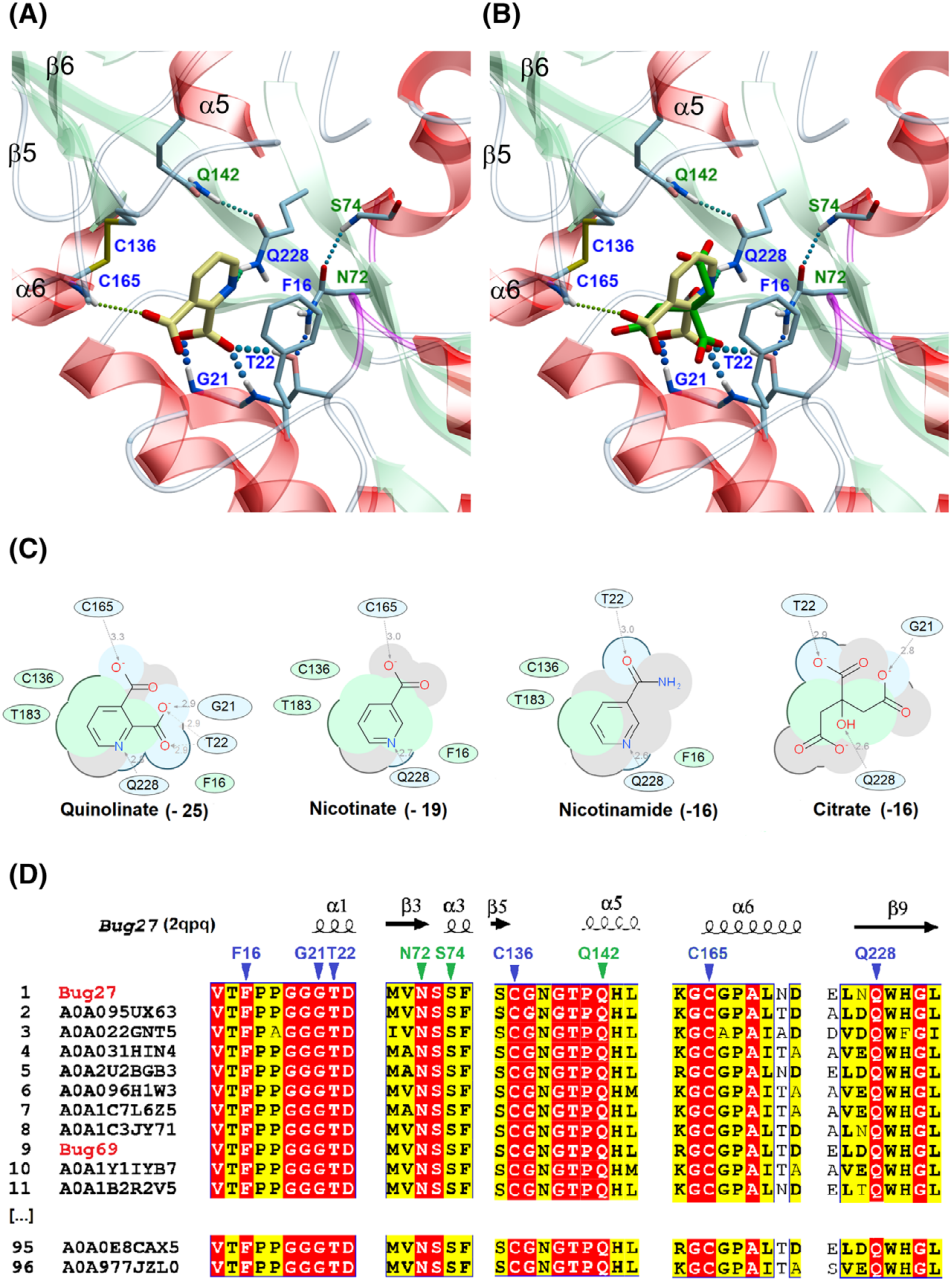
*In silico* binding analysis of Bug27. (A) Close‐up view of *Bordetella pertussis* Bug27 (PDB: 2qpq) binding site in ribbon representation with docked Qa (in yellow sticks). Secondary structure elements are color‐coded as follows: light green for β‐sheets, red for α‐helices, and white for loops or turns. Binding residues in direct contact with the ligand (with H‐bonds or hydrophobic interactions) are displayed in sticks with blue labels. Additional residues that indirectly contribute to the ligand stabilization (e.g., by forming an H‐bonding network) are displayed with green labels. H‐bonds are shown as colored dotted lines, while π‐π interactions as black dotted lines. (B) Docked Qa (yellow) overlaid with co‐crystallized citrate molecule (green) from PDB 2qpq. (C) 2D interaction diagram between tested ligands and receptor. Green and blue shading represents hydrophobic and H‐bonding contacts, respectively. Dashed arrows represent hydrogen bonds. The docking score is in brackets. The ICM software docking Score is a Generalized Born Surface Area (GBSA)/Molecular Mechanics (MM)‐type scoring function augmented with a directional hydrogen bonding term [46]. (D) Partial views of the structure‐based sequence alignment of the NAD‐related Bug27/69 TctC subfamily (Blue cluster in Fig. 2C). The alignment was performed using 96 representative sequences out of the 253 by applying an 80% sequence identity cutoff. Numbering and secondary structure elements derive from the structure of *B. pertussis* Bug27 (PDB: 2qpq). Conserved residues putatively involved in the quinolinate binding site are marked by triangles with the same color coding as panel A.

Following the *in vitro* binding study, we next performed molecular docking to get insights into the binding selectivity of Qa. After removing the non‐natural citrate ligand from the binding pocket, we used the available Bug27 (PDB: 2QPQ) structure as the template to probe Qa, Na, Nm, and citrate. In line with the *in vitro* screening and the bioinformatics predictions, Qa yielded the best scoring pose, as reflected by its most extensive interactions with the binding pocket over the other ligands (Fig. 4A–C). In detail, as shown in Fig. 4A, the carboxylic group in position 2 of Qa is firmly anchored with two hydrogen bonds to the main chain amides of Gly21 and Thr22 and with a third H‐bond to Thr22 side chain, while the carboxylate in position 3 forms an H‐bond with Cys165 (main chain). The pyridine ring is held in place by an H‐bond between the ring nitrogen and the Asn228 side chain amide and is sandwiched between Phe12 and Thr183. Additional residues, namely Ser74, Asn74, and Gln142, indirectly contribute to the ligand‐pocket H‐bonding network, while Cys136 further stabilizes the pyridine ring with a pi‐alkyl interaction (Fig. 4A). Next, we ran a structure‐based multiple sequence alignment with all members of the NAD‐related cluster previously identified with the SSN analysis. As a result, the identified ligand binding residues are universally conserved within the cluster, supporting their evolutionary selection for Qa binding in these Bug subfamilies (Fig. 4D).

**Fig. 5:**
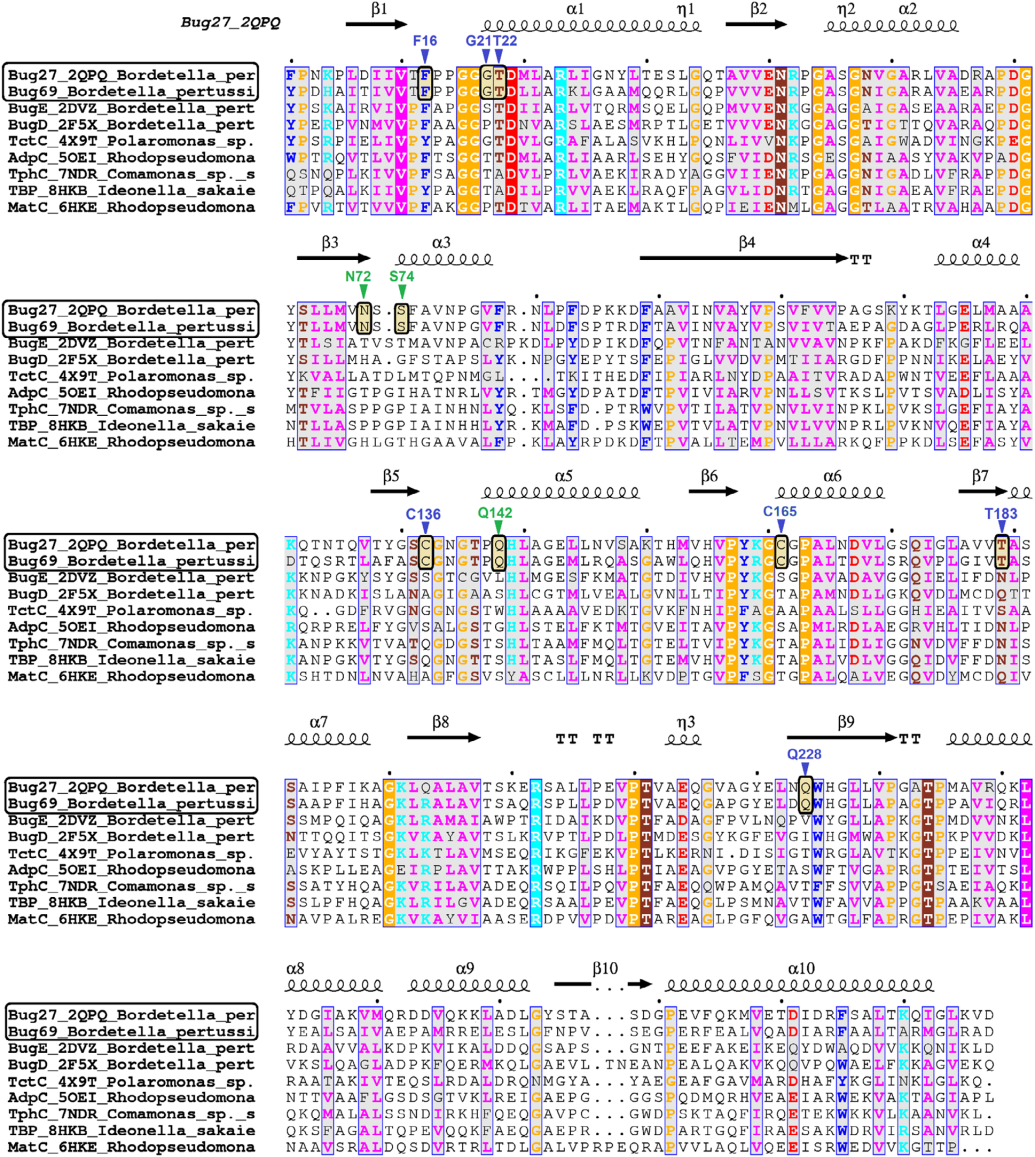
Structure‐based sequence alignment of TctC/Bug proteins with known ligand and structure. Secondary structure elements from Bug27 (PDB: 2qpq) are represented by spirals (α and 3_10_ helices) and arrows (β strands). The 3_10_ helix is labeled η. Partially conserved residues (numbered by Bug27 structure) are shown on a gray background, and universally conserved residues are highlighted by a solid background color. Triangles indicate residues identified in the quinolinic acid‐binding pocket, as also shown in Fig. 4. Highlighted boxes mark the unique conservation of such residues among the Bug27‐Bug69 pair. Ligands (in brackets) of TctC proteins used are as follows: *Bordetella pertussis* BugE (glutamate); *B. pertussis* BugD (aspartate); *Polaromonas* TctC Bpro_3516 (unknown); *Rhodopseudomonas palustris* AdpC (oxoadipate); *Comamonas* sp. TphC (terephthalate); *Ideonella sakaiensis* TBP ISF6_0226 (terephthalate); *Rhodopseudomonas palustris* MatC Rpa3494 (malate).

Along this line of thought, the Qa binding residues within the Bug69/27 family differ from their structural counterparts in other Bugs with known structures and ligands [34, 35, 36, 37, 38, 39] (Fig. 5).

## Discussion

Among the three known multicomponent transport systems that rely on the initial substrate recognition by a periplasmic binding protein, the Tripartite Tricarboxylate Transporter, prototyped by the TctABC citrate transporter system [40], is the most recent and the least characterized. Strikingly, the TTT substrate binding proteins (TctC homologs) are significantly over‐represented in some bacterial pathogens, underscoring the extensive functional diversification within this family. In addition, most *tctC* genes lack readily detectable membrane counterparts in genomic proximity, hence their assignment as “orphan” proteins. In the pathogen *B. pertussis*, only two out of 76 TctC homologs (collectively renamed as Bug proteins) have undergone a comprehensive biochemical and structural characterization: BugD and BugE, which are high‐affinity binders of aspartate and glutamate, respectively [34, 35]. Among the orphan Bug proteins, Bug27 and Bug69 have been previously associated with a pyridine transport function, but the results were preliminary. Herrou *et al*. [17] crystallized Bug27 liganded with citrate and found substantially identical affinity constants for citrate, nicotinate, and nicotinamide with a protein fluorescence quenching method. The test of Na and Nm was justified by the upregulation, among other genes, of Bug27 when bacterial cells were grown in the presence of Na. The substrate promiscuity of Bug27 and the observation that the inactivation of *bug27* did not affect Na uptake [17] prompted us to speculate that this PBP might prefer a different pyridine precursor. In parallel, Brickman et al. [16], studying the essential role of *Bordetella* NadC in the Qa salvage pathway for NAD biosynthesis, found that the gene *bug69*, which shares nearly 60% sequence identity with *bug27*, occurs in a gene cluster with *nadC* and its transcriptional regulator *nadQ* in several Bordetella species. This observation led to the assumption that Bug69 could detect Qa. Nevertheless, the genetic inactivation of Bug69 did not yield any growth defect on Qa [16], leaving the functional role of Bug69 an open question as well.

Through sequence similarity network analysis and a gene context survey across one thousand bacterial species, we identified a putative isofunctional group comprising orthologs of Bug27 and Bug69 from β‐proteobacteria. These orthologs are strongly associated with *nadC* and *nadQ*, individually or in combination, along with some clustering with other NAD‐related genes.

Thus, the most plausible hypothesis is that both Bug69 and Bug27 might participate in the transport of Qa. However, genome context analysis did not reveal a membrane domain encoded adjacent to or near Bug69 or Bug27. This is unsurprising, as Bordetella uptake genes are a disproportionately represented family of orphan SBPs, *i.e*., SBPs for which membrane transport proteins are unknown. Consequently, membrane domain partners could be shared by multiple Bugs and be encoded in distant genomic sites. Despite the strong genetic association with NAD‐related genes, other functions unrelated to NAD metabolism cannot be ruled out for Bug27 and Bug27. One possibility is that Bug27 and Bug69 are signaling proteins specialized in sensing quinolinate. This scenario could be particularly relevant in inflammation and immune response. In Eukaryotes, Qa is formed via the aerobic degradation of tryptophan (known as the kynurenine pathway) [41]. Several studies have suggested that inflammation induced by bacterial infections can lead to dysregulation of the kynurenine pathway with the concomitant increase in quinolinic acid production [42].

Through thermal shift assay, we demonstrated that Bug69 and Bug27 bind Qa with at least a 40‐fold preference over the other NAD precursors, Na and Nm. *K*_D_ values of about 0.1 μm were determined by Tryptophan fluorescence assay. These results show that Qa is an *in vitro* high‐affinity binder and the likely natural ligand of these orphan Bugs. Our results allow us to reconcile the lack of functional defect of Bug69 mutant when grown on Qa [16] due to the same function exerted by Bug27 and, possibly, other uncharacterized proteins that facilitate Qa import. We also speculate that the high concentration of Qa used in the growth medium (30 μm) might also contribute to the absence of the Bug69 mutant's defect. Indeed, a significant amount of Qa could permeate the Bordetella cells at such concentration, sustaining growth even without an import facilitator like Bug69. Similarly, *Streptococcus pyogenes* carries a similar NAD metabolism gene set as *Bordetella*, lacking *de novo* biosynthesis but retaining a functional NadC to salvage Qa from the medium [15]. This study demonstrated that *S. pyogenes* can grow on Qa as a single pyridine precursor with a minimal requirement of ~ 10 μm without an apparent Qa‐specific transporter. As in pathogenic streptococci, genome context analysis in *Bordetella pertussis* did not point to any apparent transmembrane component that could partner with Bug69 or Bug27 in Qa import. Thus, Qa can permeate the plasma membrane by diffusion or share an unidentified pyridine or dicarboxylate transporter encoded by the *B. pertussis* genome.

In conclusion, our combined experimental and bioinformatics findings suggest that *Bordetella pertussis* Bug69 and Bug27 function as high‐affinity, standalone periplasmic binding proteins of quinolinic acid. This pyridine precursor, akin to Na or Nm, can fuel NAD salvage biosynthesis in Bordetellae (Fig. 1). Our bioinformatics predictions enable confident projection of isofunctional orthologs in many β‐proteobacteria families (Fig. 2). Hopefully, this approach could be replicated to dissect the complex functional diversification of the TctC/Bug family of periplasmic binding proteins.

## Conflict of interest

The authors declare that they have no conflict of interest related to this study.

## Author contributions

LS and NR conceived and designed the project; LS, GM, and FM performed the experiments; LS performed the bioinformatics analyses; LS and GM acquired the data; LS, GM, AA, and NR analyzed and interpreted the data; LS and NR wrote the paper.

## Data Availability

The data supporting the findings of this study are available from the corresponding author, L. Sorci, at l.sorci@staff.univpm.it, upon request.

## References

[1] Scheepers GH , Lycklama ANJA and Poolman B (2016) An updated structural classification of substrate‐binding proteins. FEBS Lett 590, 4393–4401. 27714801 10.1002/1873-3468.12445

[2] Saier MH Jr (2000) A functional‐phylogenetic classification system for transmembrane solute transporters. Microbiol Mol Biol Rev 64, 354–411. 10839820 10.1128/mmbr.64.2.354-411.2000 PMC98997

[3] Eitinger T , Rodionov DA , Grote M and Schneider E (2011) Canonical and ECF‐type ATP‐binding cassette importers in prokaryotes: diversity in modular organization and cellular functions. FEMS Microbiol Rev 35, 3–67. 20497229 10.1111/j.1574-6976.2010.00230.x

[4] Rosa LT , Bianconi ME , Thomas GH and Kelly DJ (2018) Tripartite ATP‐independent periplasmic (TRAP) transporters and tripartite tricarboxylate transporters (TTT): from uptake to pathogenicity. Front Cell Infect Microbiol 8, 33. 29479520 10.3389/fcimb.2018.00033 PMC5812351

[5] Smith PC , Karpowich N , Millen L , Moody JE , Rosen J , Thomas PJ and Hunt JF (2002) ATP binding to the motor domain from an ABC transporter drives formation of a nucleotide sandwich dimer. Mol Cell 10, 139–149. 12150914 10.1016/s1097-2765(02)00576-2 PMC3516284

[6] Davies JS , Currie MJ , North RA , Scalise M , Wright JD , Copping JM , Remus DM , Gulati A , Morado DR , Jamieson SA *et al*. (2023) Structure and mechanism of a tripartite ATP‐independent periplasmic TRAP transporter. Nat Commun 14, 1120. 36849793 10.1038/s41467-023-36590-1 PMC9971032

[7] Rosa LT , Springthorpe V , Bianconi ME , Thomas GH and Kelly DJ (2018) Massive over‐representation of solute‐binding proteins (SBPs) from the tripartite tricarboxylate transporter (TTT) family in the genome of the α‐proteobacterium *Rhodoplanes* sp. Z2‐YC6860. Microbial Genomics 4, e000176. 29667925 10.1099/mgen.0.000176 PMC5994714

[8] Antoine R , Raze D and Locht C (2000) Genomics of *Bordetella pertussis* toxins. Int J Med Microbiol 290, 301–305. 11111902 10.1016/S1438-4221(00)80026-0

[9] Marinoni I , Nonnis S , Monteferrante C , Heathcote P , Härtig E , Böttger LH , Trautwein AX , Negri A , Albertini AM and Tedeschi G (2008) Characterization of l‐aspartate oxidase and quinolinate synthase from *Bacillus subtilis* . FEBS J 275, 5090–5107. 18959769 10.1111/j.1742-4658.2008.06641.x

[10] Sharma V , Grubmeyer C and Sacchettini JC (1998) Crystal structure of quinolinic acid phosphoribosyltransferase from *Mmycobacterium tuberculosis*: a potential TB drug target. Structure 6, 1587–1599. 9862811 10.1016/s0969-2126(98)00156-7

[11] Sorci L , Kurnasov O , Rodionov D and Osterman A (2010) Genomics and enzymology of NAD biosynthesis. In Comprehensive Natural Products II: Chemistry and Biology, Vol 7 ( Lew M and Hung‐Wen L , eds), pp. 213–257. Elsevier, Oxford.

[12] Rodionov DA , Li X , Rodionova IA , Yang C , Sorci L , Dervyn E , Martynowski D , Zhang H , Gelfand MS and Osterman AL (2008) Transcriptional regulation of NAD metabolism in bacteria: genomic reconstruction of NiaR (YrxA) regulon. Nucleic Acids Res 36, 2032–2046. 18276644 10.1093/nar/gkn046 PMC2330245

[13] McPheat WL and Wardlaw AC (1980) Uptake of [14C]nicotinic acid and [14C]nicotinamide by Bordetella pertussis. FEMS Microbiol Lett 7, 341–343.

[14] Prunier AL , Schuch R , Fernandez RE and Maurelli AT (2007) Genetic structure of the nadA and nadB antivirulence loci in shigella spp. J Bacteriol 189, 6482–6486. 17586625 10.1128/JB.00525-07 PMC1951923

[15] Sorci L , Blaby IK , Rodionova IA , De Ingeniis J , Tkachenko S , de Crecy‐Lagard V and Osterman AL (2013) Quinolinate salvage and insights for targeting NAD biosynthesis in group a streptococci. J Bacteriol 195, 726–732. 23204464 10.1128/JB.02002-12 PMC3562111

[16] Brickman TJ , Suhadolc RJ , McKelvey PJ and Armstrong SK (2017) Essential role of *Bordetella* NadC in a quinolinate salvage pathway for NAD biosynthesis. Mol Microbiol 103, 423–438. 27783449 10.1111/mmi.13566

[17] Herrou J , Bompard C , Antoine R , Leroy A , Rucktooa P , Hot D , Huvent I , Locht C , Villeret V and Jacob‐Dubuisson F (2007) Structure‐based mechanism of ligand binding for periplasmic solute‐binding protein of the bug family. J Mol Biol 373, 954–964. 17870093 10.1016/j.jmb.2007.08.006

[18] Laemmli U (1970) SDS‐page Laemmli method. Nature 227, 680–685. 5432063 10.1038/227680a0

[19] Bradford MM (1976) A rapid and sensitive method for the quantitation of microgram quantities of protein utilizing the principle of protein‐dye binding. Anal Biochem 72, 248–254. 942051 10.1016/0003-2697(76)90527-3

[20] Pantoliano MW , Petrella EC , Kwasnoski JD , Lobanov VS , Myslik J , Graf E , Carver T , Asel E , Springer BA , Lane P *et al*. (2001) High‐density miniaturized thermal shift assays as a general strategy for drug discovery. J Biomol Screen 6, 429–440. 11788061 10.1177/108705710100600609

[21] Liu X , Obianyo O , Chan CB , Huang J , Xue S , Yang JJ , Zeng F , Goodman M and Ye K (2014) Biochemical and biophysical investigation of the brain‐derived neurotrophic factor mimetic 7,8‐dihydroxyflavone in the binding and activation of the TrkB receptor. J Biol Chem 289, 27571–27584. 25143381 10.1074/jbc.M114.562561 PMC4183797

[22] Shannon P , Markiel A , Ozier O , Baliga NS , Wang JT , Ramage D , Amin N , Schwikowski B and Ideker T (2003) Cytoscape: a software environment for integrated models of biomolecular interaction networks. Genome Res 13, 2498–2504. 14597658 10.1101/gr.1239303 PMC403769

[23] Abagyan R , Totrov M and Kuznetsov D (1994) ICM—A new method for protein modeling and design: applications to docking and structure prediction from the distorted native conformation. J Comput Chem 15, 488–506.

[24] An J , Totrov M and Abagyan R (2005) Pocketome via comprehensive identification and classification of ligand binding envelopes. Mol Cell Proteomics 4, 752–761. 15757999 10.1074/mcp.M400159-MCP200

[25] Pei J , Kim BH and Grishin NV (2008) PROMALS3D: a tool for multiple protein sequence and structure alignments. Nucleic Acids Res 36, 2295–2300. 18287115 10.1093/nar/gkn072 PMC2367709

[26] Gouet P , Robert X and Courcelle E (2003) ESPript/ENDscript: extracting and rendering sequence and 3D information from atomic structures of proteins. Nucleic Acids Res 31, 3320–3323. 12824317 10.1093/nar/gkg556 PMC168963

[27] Oberg N , Zallot R and Gerlt JA (2023) EFI‐EST, EFI‐GNT, and EFI‐CGFP: enzyme function initiative (EFI) web resource for genomic enzymology tools. J Mol Biol 435, 168018. 37356897 10.1016/j.jmb.2023.168018 PMC10291204

[28] Zallot R , Oberg N and Gerlt JA (2019) The EFI web resource for genomic enzymology tools: leveraging protein, genome, and metagenome databases to discover novel enzymes and metabolic pathways. Biochemistry 58, 4169–4182. 31553576 10.1021/acs.biochem.9b00735 PMC7057060

[29] Gerlt JA (2017) Genomic enzymology: web tools for leveraging protein family sequence‐function space and genome context to discover novel functions. Biochemistry 56, 4293–4308. 28826221 10.1021/acs.biochem.7b00614 PMC5569362

[30] Atkinson HJ , Morris JH , Ferrin TE and Babbitt PC (2009) Using sequence similarity networks for visualization of relationships across diverse protein superfamilies. PLoS One 4, e4345. 19190775 10.1371/journal.pone.0004345 PMC2631154

[31] Minazzato G , Gasparrini M , Heroux A , Sernova NV , Rodionov DA , Cianci M , Sorci L and Raffaelli N (2022) Bacterial NadQ (COG4111) is a Nudix‐like, ATP‐responsive regulator of NAD biosynthesis. J Struct Biol 214, 107917. 36332744 10.1016/j.jsb.2022.107917

[32] Hove‐Jensen B , Brodersen DE and Manav MC (2019) The prodigal compound: return of ribosyl 1,5‐bisphosphate as an important player in metabolism. Microbiol Mol Biol Rev 83, e00040‐18. 30567937 10.1128/MMBR.00040-18 PMC6383446

[33] Hove‐Jensen B , Rosenkrantz TJ , Haldimann A and Wanner BL (2003) Escherichia coli phnN, encoding ribose 1,5‐bisphosphokinase activity (phosphoribosyl diphosphate forming): dual role in phosphonate degradation and NAD biosynthesis pathways. J Bacteriol 185, 2793–2801. 12700258 10.1128/JB.185.9.2793-2801.2003 PMC154390

[34] Huvent I , Belrhali H , Antoine R , Bompard C , Locht C , Jacob‐Dubuisson F and Villeret V (2006) Crystal structure of *Bordetella pertussis* BugD solute receptor unveils the basis of ligand binding in a new family of periplasmic binding proteins. J Mol Biol 356, 1014–1026. 16403514 10.1016/j.jmb.2005.11.096

[35] Huvent I , Belrhali H , Antoine R , Bompard C , Locht C , Jacob‐Dubuisson F and Villeret V (2006) Structural analysis of *Bordetella pertussis* BugE solute receptor in a bound conformation. Acta Crystallogr D Biol Crystallogr 62, 1375–1381. 17057341 10.1107/S0907444906032653

[36] Rosa LT , Dix SR , Rafferty JB and Kelly DJ (2017) Structural basis for high‐affinity adipate binding to AdpC (RPA4515), an orphan periplasmic‐binding protein from the tripartite tricarboxylate transporter (TTT) family in *Rhodopseudomonas palustris* . FEBS J 284, 4262–4277. 29082669 10.1111/febs.14304

[37] Gautom T , Dheeman D , Levy C , Butterfield T , Alvarez Gonzalez G , Le Roy P , Caiger L , Fisher K , Johannissen L and Dixon N (2021) Structural basis of terephthalate recognition by solute binding protein TphC. Nat Commun 12, 6244. 34716322 10.1038/s41467-021-26508-0 PMC8556258

[38] Lee SH , Seo H , Hong H , Kim M and Kim K‐J (2023) Molecular mechanism underlying high‐affinity terephthalate binding and conformational change of TBP from Ideonella sakaiensis. Int J Biol Macromol 243, 125252. 37295700 10.1016/j.ijbiomac.2023.125252

[39] Rosa LT , Dix SR , Rafferty JB and Kelly DJ (2019) A new mechanism for high‐affinity uptake of C4‐dicarboxylates in bacteria revealed by the structure of *Rhodopseudomonas palustris* MatC (RPA3494), a periplasmic binding protein of the tripartite tricarboxylate transporter (TTT) family. J Mol Biol 431, 351–367. 30471256 10.1016/j.jmb.2018.11.016

[40] Winnen B , Hvorup RN and Saier MH Jr (2003) The tripartite tricarboxylate transporter (TTT) family. Res Microbiol 154, 457–465. 14499931 10.1016/S0923-2508(03)00126-8

[41] Ruggieri S , Orsomando G , Sorci L and Raffaelli N (2015) Regulation of NAD biosynthetic enzymes modulates NAD‐sensing processes to shape mammalian cell physiology under varying biological cues. Biochim Biophys Acta, Proteins Proteomics 1854, 1138–1149. 10.1016/j.bbapap.2015.02.021 25770681

[42] Mandi Y , Stone TW , Guillemin GJ , Vecsei L and Williams RO (2022) Editorial: multiple implications of the kynurenine pathway in inflammatory diseases: diagnostic and therapeutic applications. Front Immunol 13, 860867. 35251052 10.3389/fimmu.2022.860867 PMC8892578

[43] Rodionova IA , Zuccola HJ , Sorci L , Aleshin AE , Kazanov MD , Ma CT , Sergienko E , Rubin EJ , Locher CP and Osterman AL (2015) Mycobacterial nicotinate mononucleotide adenylyltransferase: structure, mechanism, and implications for drug discovery. J Biol Chem 290, 7693–7706. 25631047 10.1074/jbc.M114.628016 PMC4367272

[44] LaRonde‐LeBlanc N , Resto M and Gerratana B (2009) Regulation of active site coupling in glutamine‐dependent NAD(+) synthetase. Nat Struct Mol Biol 16, 421–429. 19270703 10.1038/nsmb.1567

[45] Sorci L , Brunetti L , Cialabrini L , Mazzola F , Kazanov MD , D'Auria S , Ruggieri S and Raffaelli N (2014) Characterization of bacterial NMN deamidase as a ser/Lys hydrolase expands diversity of serine amidohydrolases. FEBS Lett 588, 1016–1023. 24530526 10.1016/j.febslet.2014.01.063

[46] Neves MA , Totrov M and Abagyan R (2012) Docking and scoring with ICM: the benchmarking results and strategies for improvement. J Comput Aided Mol Des 26, 675–686. 22569591 10.1007/s10822-012-9547-0 PMC3398187

